# Preparing the Younger Generation for an Aging Society: Strategies, Challenges, and Opportunities

**DOI:** 10.7759/cureus.64121

**Published:** 2024-07-09

**Authors:** Nor Faiza Mohd. Tohit, Mainul Haque

**Affiliations:** 1 Department of Community Health, Universiti Pertahanan Nasional Malaysia (National Defence University of Malaysia), Kuala Lumpur, MYS; 2 Karnavati Scientific Research Center (KSRC), School of Dentistry, Karnavati University, Gandhinagar, IND; 3 Department of Pharmacology and Therapeutics, National Defence University of Malaysia, Kuala Lumpur, MYS

**Keywords:** organizing younger cohort, approaches contests and openings, age-friendly society, empathy development, policy interventions, community engagement, youth education, aging population, demographic shift, ageism

## Abstract

The global demographic landscape is experiencing a monumental shift as populations age, driven by advances in healthcare and declining birth rates. This transition underscores the need to prepare the younger generation to navigate and contribute effectively to an aging society. This manuscript comprehensively reviews strategies to equip younger generations with the requisite knowledge, skills, and empathy to support an aging population. This study identifies critical challenges and opportunities in fostering intergenerational solidarity and understanding through an extensive analysis of existing literature and innovative educational programs. The review highlights the importance of early education, community engagement, and policy interventions in bridging the generational divide. Additionally, it explores the role of technology and digital media in facilitating awareness and empathy among young people. Key findings suggest that incorporating aging-related content into educational curricula, promoting volunteerism, and implementing supportive policies can significantly enhance the younger generation’s readiness to support an aging society. The manuscript concludes with recommendations for future research and practical steps for educators, policymakers, and community leaders to foster a more inclusive and age-friendly environment. By preparing the younger generation today, we can build a more cohesive and supportive society for tomorrow.

## Introduction and background

The global demographic landscape is undergoing a significant transformation, characterized by an increasing proportion of elderly individuals relative to younger populations. This shift, often referred to as population aging, presents profound socio-economic, political, and cultural challenges and opportunities [1]. As life expectancy rises and birth rates decline, societies worldwide must adapt to ensure sustainable development and well-being for all ages [2,3]. Therefore, preparing the younger generation to meet the needs of an aging society has become a critical priority.

An aging population presents numerous challenges that impact various aspects of society. One of the primary challenges is the increased demand for healthcare services [4,5]. As individuals age, they typically experience a higher incidence of chronic illnesses and require more frequent medical attention. This heightened demand can strain healthcare systems, leading to longer times, higher costs, and a need for more healthcare professionals [6]. Additionally, the financial burden associated with aging populations is significant. Increased healthcare costs, pension liabilities, and social security expenditures can pressure public finances considerably, potentially leading to higher taxes or reduced benefits for other societal groups [7-9].

Moreover, the workforce is affected as the proportion of elderly individuals grows. This demographic shift can lead to a shrinking labor force, which may result in lower economic productivity and potential skill shortages in crucial sectors [10,11]. Employers may face challenges retaining experienced workers while ensuring opportunities for younger employees to advance. Another significant challenge is the rise in caregiving needs. As the population ages, there is a more substantial requirement for long-term care services, both formal and informal [12]. Families often bear the brunt of this responsibility, leading to financial strain, emotional stress, and reduced work productivity among caregivers [13,14].

**Figure 1:**
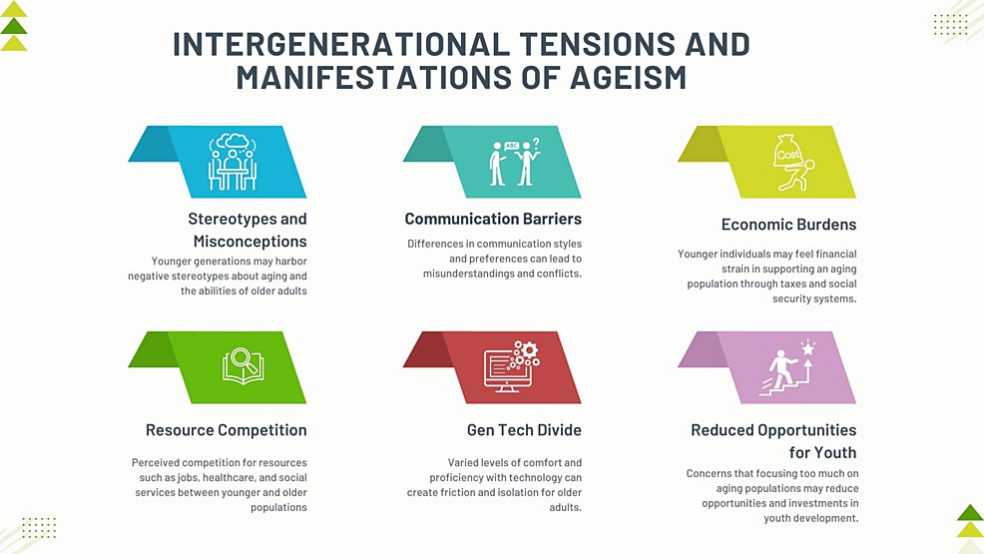
Intergenerational tension and manifestations of ageism Image credit: Nor Faiza Mohd. Tohit

Social challenges also accompany an aging population. Ageism and intergenerational tensions are additional concerns that must be addressed to foster a harmonious, inclusive society [15,16]. Levy describes ageism as prejudice or discrimination against individuals based on their age, which can impede social cohesion and marginalize older adults [17]. Ageism and intergenerational tensions can become more pronounced, as younger individuals may harbor misconceptions about aging or feel burdened by the economic implications of supporting older generations [17]. The manifestation of ageism and integrational tension is shown in Figure 1.

Addressing these attitudes is essential for fostering a cohesive society where all age groups are valued and respected. Additionally, ensuring the social inclusion and active participation of older adults in community life is vital to prevent isolation and maintain their mental health and well-being [18,19]. An adequately prepared younger generation, educated in gerontology, healthcare, and social work, is essential to addressing these emerging needs [20]. Educational institutions play a pivotal role in equipping young people with the knowledge and skills needed to support an aging population through curricula that include gerontological education and practical training in eldercare. Research indicates that intergenerational programs promote interaction and understanding between different age groups and can mitigate ageist attitudes among young people and build empathetic, supportive relationships [21,22]. These programs often involve collaborative projects, shared learning experiences, and community service initiatives that bridge generational gaps [23,24].

Technological advancements hold promise for enhancing intergenerational solidarity and supporting aging populations. Innovations such as telemedicine, assistive technologies, and communication platforms can improve the quality of life for older adults while providing valuable learning and career opportunities for younger individuals trained in these fields [25-27]. Preparing youth for careers in technology-driven eldercare can address workforce shortages and ensure that emerging needs are met with innovative solutions. Cultural and socio-economic factors shape the success of efforts to prepare the younger generation for an aging society. Socio-economic disparities, cultural norms, and family structures influence attitudes toward aging and the willingness to engage in intergenerational initiatives [28,29]. Understanding these contextual factors is crucial for developing effective strategies that resonate with diverse communities.

**Figure 2:**
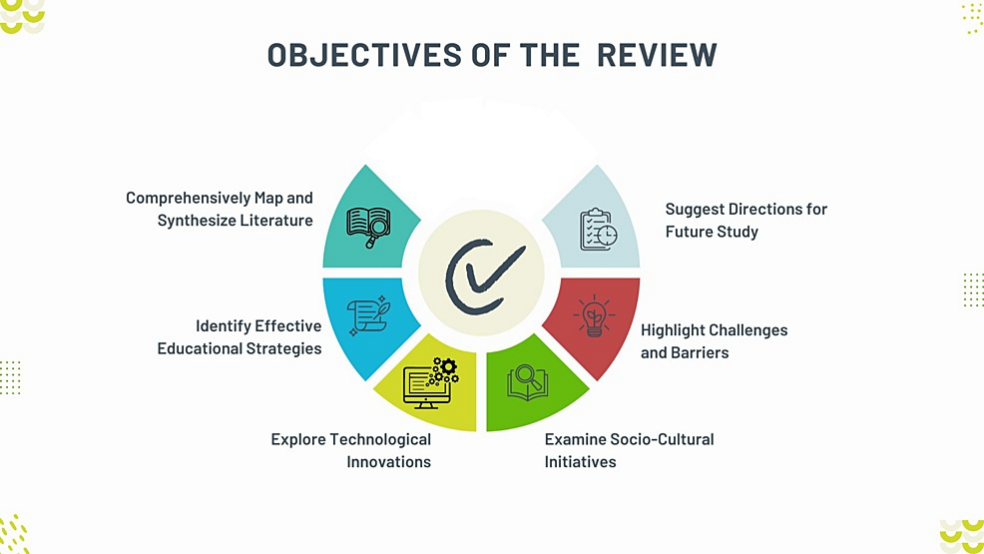
Objectives of the scoping review Image credit: Nor Faiza Mohd. Tohit

This scoping review aims to comprehensively map and synthesize the existing literature on strategies, challenges, and opportunities for preparing the younger generation for an aging society. Specifically, this review aims to identify effective educational strategies that equip young people with the necessary skills and knowledge to support and engage with an aging population. It also seeks to explore technological innovations that facilitate intergenerational solidarity and improve the quality of life for older adults. Additionally, the review will examine socio-cultural initiatives that foster intergenerational understanding and reduce ageism. It highlights the challenges and barriers faced in implementing these strategies and initiatives. Furthermore, the review will identify gaps in the current research and suggest directions for future study. By achieving these objectives, as outlined in Figure 2, the review seeks to provide a comprehensive overview of best practices and actionable insights to inform policymakers, educators, and communities in designing effective interventions to support an aging society.

## Review

Methods and materials

**Figure 3:**
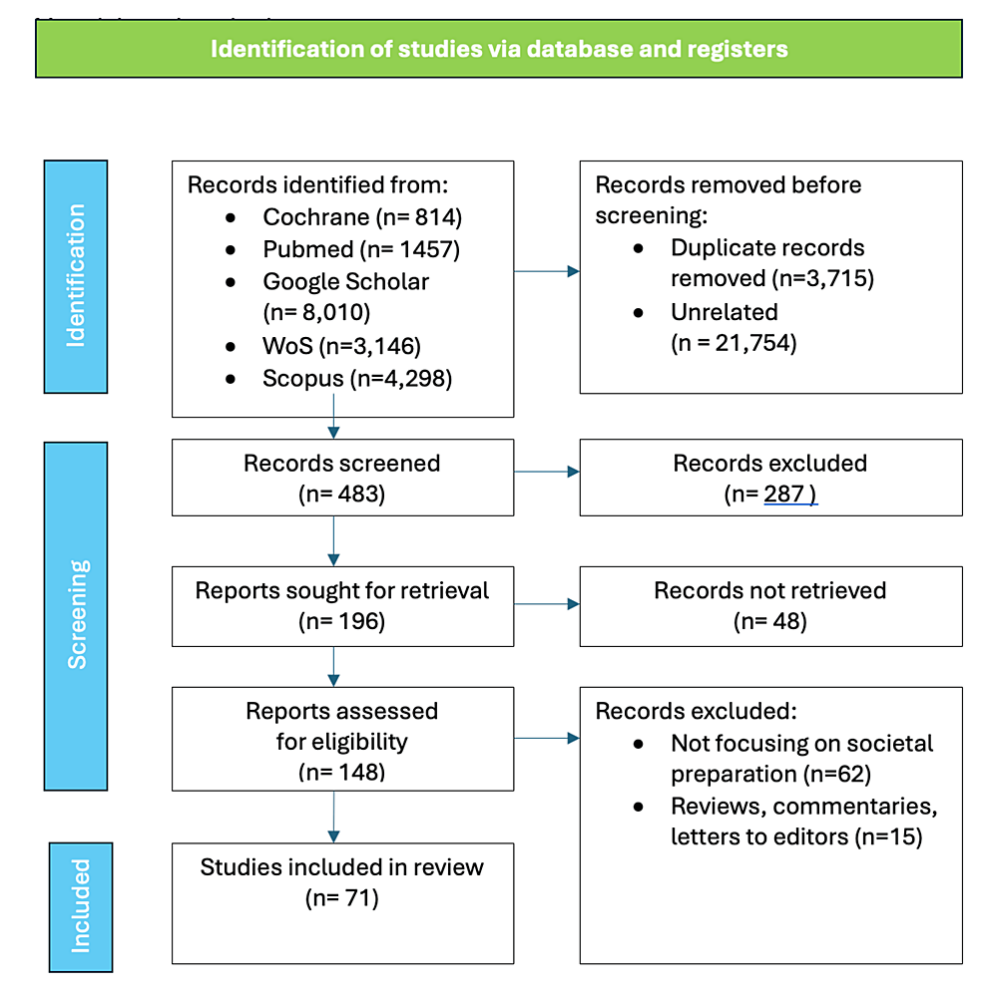
Process flow of conducting the review Image credit: Nor Faiza Mohd. Tohit

To conduct a comprehensive scoping review on “Preparing the Younger Generation for an Aging Society: Strategies, Challenges, and Opportunities,” we followed the methodological framework developed by Arksey and O'Malley [30] and further refined by Levac et al. [31]. This framework involves five key steps: identifying the research question, relevant studies, study selection, charting the data, and collating, summarizing, and reporting the results. Figure 3 depicts the process of conducting this review.

Identifying the Research Question

The primary research question guiding this scoping review is: What are the effective strategies, challenges, and opportunities in preparing the younger generation for an aging society? Secondary questions include identifying current educational practices, technological interventions, and socio-cultural factors impacting intergenerational solidarity.

Identifying Relevant Studies

A systematic search was conducted across multiple databases, including PubMed, Scopus, Cochrane, and Web of Science, covering literature published from January 2014 to May 2024. Search terms included combinations of keywords and MeSH terms such as “intergenerational programs,” AND “ageism,” AND “aging education,” AND “youth and elderly interaction,” AND “social cohesion,” AND “technology in eldercare,” AND “gerontology education.” The search was supplemented by manual searches of reference lists of relevant articles and gray literature, such as reports from aging and youth education organizations.

Study Selection

The inclusion criteria were peer-reviewed articles, reviews, and reports focusing on strategies to prepare youth for aging societies, including educational interventions, technological solutions, and socio-cultural programs. These inclusion criteria are crucial for defining the scope of the review and guiding the identification and selection of relevant literature for the scoping review. Studies were excluded if they did not address intergenerational aspects, were not available in English, or were editorials and opinion pieces without empirical data. The studies that primarily focus on the older adult population without direct implications for preparing the younger generation for an aging society were also excluded. The focus is on the younger generation’s preparation and engagement with an aging society. The studies that do not directly relate to the theme of the scoping review, such as studies solely focused on clinical interventions for specific age-related conditions that are not associated with the overarching societal preparation for an aging population, were also excluded.

Charting the Data

Data were extracted and charted using a standardized form capturing critical information: authorship, publication year, country of study, objectives, methodology, population, interventions or strategies, outcomes, and identified challenges and opportunities. This form ensured consistency and comprehensive coverage of relevant data.

Collating, Summarizing, and Reporting the Results

The extracted data were synthesized to highlight prevalent themes, effective strategies, and notable barriers. The synthesis was organized into three main sections: educational strategy, technological innovations, and socio-cultural initiatives. It focused on identifying challenges and proposing actionable opportunities for future implementation. This comprehensive approach thoroughly explored existing literature, providing a solid foundation for understanding the multifaceted aspects of preparing the younger generation for an aging society.

Review of literature

Advantages of Preparing the Younger Generation for an Aging Society

Preparing the younger generation to understand and support an aging society yields a multitude of benefits that enhance social cohesion, economic stability, and overall societal well-being. One of the primary advantages is the reduction of ageism, which is often rooted in misconceptions and stereotypes about aging [15-17,32]. Educational programs that include aging studies and intergenerational activities have been shown to reduce prejudices and foster empathy among young people [21-24]. Research indicates that such initiatives can lead to lasting changes in attitudes, ultimately cultivating a more inclusive and respectful society [33].

Another significant benefit is the enhancement of intergenerational solidarity. Programs that promote interaction between younger and older individuals, such as community projects, shared learning experiences, and mentoring schemes, help bridge generational divides [34,35]. These interactions provide young people with valuable life skills and wisdom from older adults and counteract the social isolation often experienced by the elderly. By fostering mutual understanding and respect, these programs create a more cohesive and connected community [36]. Moreover, equipping the younger generation with skills relevant to eldercare, healthcare, and social services addresses critical workforce shortages in these essential sectors. As the demand for healthcare services and long-term care increases with an aging population, having a well-prepared workforce is vital [10,11,37]. Educational institutions and policymakers can support this by incorporating gerontology and caregiving into curricula [38-40] and providing incentives for careers in these fields [41,42]. This ensures that older populations receive high-quality care and support, which is crucial for their well-being.

**Figure 4:**
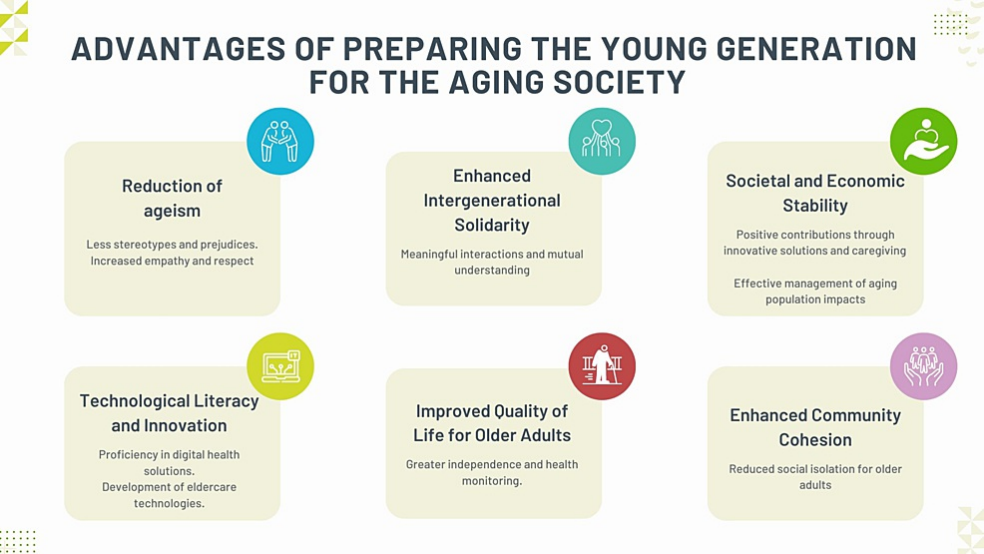
The advantages of preparing the young generation for the aging society Image credit: Nor Faiza Mohd. Tohit

Technological literacy among young people also plays a crucial role in supporting an aging society. As technology becomes increasingly integral to eldercare, the younger generation must be adept at using and developing digital health solutions. Innovations such as telehealth, wearable health devices, and smart home technologies can significantly improve the quality of life for older adults by providing them with greater independence, better health monitoring, and easier access to medical services [43, 44]. Encouraging science, technology, engineering, and mathematics (STEM) education and fostering technological innovation can thus create a generation that supports older adults and drives advancements that benefit all age groups [45-48]. Fostering a culture that values and supports older adults contributes to societal and economic stability. Communities prioritizing intergenerational collaboration and support structures are better equipped to manage an aging population’s financial and social implications [28,29]. Young people who are educated about the dynamics and challenges of aging are more likely to contribute positively to these efforts, whether through innovative solutions, compassionate caregiving, or active participation in policy advocacy [45,49,50]. This holistic approach ensures all generations’ sustainable and equitable future [51]. The advantages of preparing the young generation for the aging society are shown in Figure 4.

Strategies to Equip the Young Generation for an Aging Society

Educational strategies: Integrating aging education into school curricula is a pivotal strategy for preparing the younger generation to contribute positively to an aging society. Educating students about the biological, psychological, and social aspects of aging can foster a deeper understanding and appreciation of the elderly. Curricular content might include gerontology, the aging process, the demographic shift’s societal implications, and the challenges and opportunities associated with aging populations. Studies suggest that early exposure to aging-related topics can positively influence youths’ attitudes toward older adults and reduce ageism [21-24]. Experiential learning is an effective method for developing empathy and understanding among young people regarding the elderly. This approach involves direct engagement and interaction with older adults, which humanizes aging and breaks stereotypes. Programs like intergenerational mentorships, youth volunteering at senior centers, and school visits to nursing homes allow students to form meaningful connections with elderly individuals [52]. Experiential learning has been shown to significantly enhance empathy, improve communication skills, and foster a more compassionate outlook towards the older population [34]

Several educational programs have successfully integrated aging education and experiential learning to prepare youths for an aging society. For instance, Japan’s “Aging Awareness and Employment Program” includes curriculum modules on aging and practical sessions where students interact with elderly community members [53]. Another notable example is the “Generations Together” program in the United States, which combines classroom instruction with community service, allowing students to work directly with older adults on various projects [54]. These programs have substantially improved students’ attitudes toward aging and increased their social responsibility toward older generations. Educators can effectively prepare the younger generation to navigate and support an aging society by incorporating aging education, promoting experiential learning, and examining successful case studies. These methods equip young people with the necessary knowledge and skills and cultivate a more inclusive, empathetic, and age-friendly community.

Community engagement and intergenerational programs: Community involvement is essential for preparing younger generations to support an aging society. Engaging youth in community activities that involve older adults helps bridge the generational gap, fostering mutual respect, understanding, and cooperation. Community-based programs provide practical opportunities for young people to learn about aging in a real-world context, making theoretical knowledge more concrete and impactful. Participating in these initiatives, young people develop a sense of social responsibility and civic engagement, which is crucial for building a cohesive and supportive society. Research has shown that communities that actively encourage intergenerational interaction tend to have more robust social networks and reduced social isolation among older adults [55,56]. Several successful intergenerational projects demonstrate the positive impact of these initiatives. One prominent example is the “Adopt-a-Grandparent” program, where students are paired with elderly members of the community for regular visits and shared activities. This program has been shown to improve the emotional well-being of older adults and increase empathy and social skills among students [57]. Another example is the “Intergenerational School” in Cleveland, OH, which integrates elderly volunteers into the classroom to work alongside students. This unique educational model has improved academic performance and a greater sense of community among students while providing older adults with a sense of purpose and continued engagement [5,58].

Fostering intergenerational relationships offers numerous benefits to both young people and older adults. For youth, these interactions promote emotional intelligence, empathy, and respect for the elderly while enhancing their understanding of aging. Young people in intergenerational programs often report a more positive attitude toward aging and a more substantial commitment to community service [35]. Intergenerational engagement combats loneliness and social isolation, improving the geriatric community’s mental and emotional health. It allows them to share their knowledge and experiences, fostering a sense of value and purpose. Communities benefit, as intergenerational harmony strengthens social cohesion and builds a more inclusive society [18,19]. By emphasizing community involvement through intergenerational projects, society can harness the strengths and resources of both young and older generations, creating a mutually beneficial environment that promotes understanding, respect, and solidarity.

Policy and advocacy: Preparing the younger generation effectively for an aging society requires strategic policy-making and robust advocacy efforts. Educational policies must prioritize the integration of aging and intergenerational topics into school curricula at all levels. This can include formal education on aging processes, the socio-economic impacts of an aging population, and practical training in gerontology and eldercare [59]. Incorporating such content helps cultivate empathy, reduce ageism, and equip young people with the skills needed to support an aging society [15,16]. Policies should also encourage and support intergenerational programs that foster meaningful interactions between younger and older generations. These programs can be implemented in schools, community organizations, and workplaces, promoting mutual understanding and solidarity [20,35,52]. Governments and educational institutions can collaborate to provide incentives and resources for these initiatives, ensuring their sustainability and effectiveness.

Technological literacy is another critical area where policy interventions are necessary. As technology increasingly assists in eldercare through telehealth, digital health platforms, and assistive devices, the younger generation must be well-versed in these technologies. Policies should promote STEM education and invest in technological infrastructure that benefits young and older populations [25-27,46]. Advocacy efforts must also focus on reshaping societal attitudes toward aging. Campaigns to raise awareness about the value of older adults and combat ageist stereotypes are crucial. Advocacy groups can work with media organizations, educational institutions, and community leaders to disseminate positive narratives about aging and the contributions of older individuals [5,10,58]. Furthermore, concerted efforts must address the economic aspects of an aging society. Policies should support gerontology, healthcare, and social work career pathways, ensuring these fields are attractive and accessible to young people [60]. Financial incentives, scholarships, and clear career progression paths can help attract and retain young professionals in these critical areas [41,42]. Preparing the younger generation for an aging society requires a multifaceted approach involving educational reform, technological investment, intergenerational initiatives, and proactive advocacy to foster an inclusive and supportive environment for all age groups.

Technological and innovative solutions: Utilizing technology is a powerful method to bridge the generational gap and prepare youth for an aging society. Modern technological advancements provide tools that facilitate communication and understanding between generations. Virtual reality (VR) and augmented reality (AR) can simulate aging-related conditions, helping young people to empathize with the elderly by experiencing visual impairments, mobility challenges, and other age-related issues [60,61]. These immersive experiences foster a deeper understanding and dispel stereotypes about aging. Additionally, digital storytelling platforms allow older adults to share their life stories and wisdom, enabling young people to learn from their experiences and cultivate a greater appreciation for their perspectives [62].

Technological innovations in caregiving and elderly support systems have significantly enhanced the quality of life for older adults while offering learning opportunities for younger generations. Smart home technologies, such as IoT devices, can monitor the health and safety of elderly individuals, providing real-time alerts to caregivers for timely interventions [63]. Wearable health devices track vital signs, ensuring continuous health monitoring and enabling preventive care. Robotics, including social robots like companion pets, have been developed to offer emotional support and reduce loneliness among older adults, engaging youth in technology-driven caregiving approaches [64,65]. Social media and digital platforms foster awareness about aging and promote intergenerational solidarity. Campaigns and educational content on platforms like Facebook, Instagram, and TikTok inform young audiences about the realities and challenges of aging [66]. Online forums and communities facilitate conversations between different age groups, allowing young people to connect with and learn from older adults. Moreover, e-learning courses focused on gerontology and elder care available on MOOC platforms like Coursera and edX enable young people to gain knowledge and skills relevant to supporting an aging population [67]. We can effectively prepare younger generations to support and thrive in an aging society by leveraging technology, innovative caregiving solutions, and digital platforms. These strategies enhance understanding and empathy and encourage active participation in fostering an inclusive and supportive community for all ages [68].

Overcoming Challenges and Barriers in Preparing Youth for an Aging Society

Socio-economic and cultural barriers to intergenerational solidarity: Socio-economic and cultural barriers significantly impede the efforts to foster intergenerational solidarity. From a socio-economic perspective, income inequality, disparate access to educational resources, and economic instability can strain relationships between different age groups. Financial challenges may lead to competition for limited resources and opportunities, creating a divide rather than promoting cooperation. For instance, research shows that economic insecurity can exacerbate generational tensions, with young people often feeling that they bear the financial burdens of supporting an aging population through taxes and social security contributions [69,70]. Culturally, generations’ differing values, beliefs, and lifestyles can also hinder intergenerational understanding. Older adults may hold onto traditional norms and practices, while younger generations seek more progressive and technologically driven lifestyles [28,29]. These cultural differences can contribute to misunderstandings and reduced empathy between age groups. Additionally, in many cultures, the rapid pace of technological change has widened the digital divide, further estranging older and younger generations [71].

Addressing ageism and stereotypes: Addressing ageism and stereotypes is crucial in promoting empathy and understanding between generations. Ageism, which involves discrimination based on a person’s age, is pervasive and affects both younger and older individuals. It manifests in various forms, including workplace discrimination, social exclusion, and negative portrayals in media. Younger individuals often internalize these stereotypes, leading to biased attitudes towards older adults [21-24]. Educational interventions are essential in combating ageism. Programs that include aging education in school curricula can dispel myths and stereotypes about aging, promoting a more nuanced understanding of aging [17]. Empathy-building activities, such as role-playing and intergenerational dialogue, can help students appreciate the lived experiences of older adults. Studies have shown that such interventions reduce ageist attitudes and foster mutual respect [33,36]. Moreover, media campaigns that challenge ageist stereotypes and highlight the contributions of older adults to society can shift public perceptions. These campaigns can showcase older individuals as active, engaged, and valuable community members, thus altering the narrative around aging [52].

Overcoming resistance to change in educational systems: Educational systems play a pivotal role in shaping the attitudes and values of the younger generation. However, resistance to change within these systems can hinder the integration of aging education and intergenerational programs. Traditional curricula often lack content on aging, and educators may be reluctant to adopt new teaching methods or materials. To overcome this resistance, it is crucial to demonstrate the value of aging education through evidence-based research and pilot programs. Collaboration with educational stakeholders, including teachers, administrators, and policymakers, can facilitate the gradual integration of aging-related topics [34,36]. For example, pilot programs that successfully incorporate intergenerational activities into the curriculum can serve as models for broader adoption [36,37]. Professional development and training for educators are also critical. Teachers must have the knowledge and skills to deliver aging education and effectively facilitate intergenerational interactions. Workshops, seminars, and continuing education courses can help educators understand the importance of this content and how to integrate it into their teaching practices [47,49]. Furthermore, involving students in the process of curricular change can amplify their voices and foster a sense of ownership. Participatory approaches that engage students in designing and implementing intergenerational programs can enhance their commitment to and enthusiasm for these initiatives [21-23]. Addressing socio-economic and cultural barriers, combating ageism and stereotypes, and overcoming resistance to change in educational systems are essential strategies for preparing youth for an aging society. Understanding and addressing these challenges can create a more inclusive, empathetic, and supportive community where younger and older generations thrive.

Opportunities for the Future

Potential benefits of a well-prepared younger generation: A well-prepared younger generation capable of addressing the needs of an aging society presents numerous benefits. First, equipping youth with knowledge about aging and eldercare can lead to a more empathetic and compassionate society. Young people who understand the challenges and experiences of older adults are more likely to advocate for policies and practices that support aging populations [21-24]. Educational programs incorporating gerontology and intergenerational projects can foster these attitudes, reducing ageism and increasing social cohesion [33-35]. Moreover, preparing youth for careers in senior healthcare, social work, and elder law can address the increasing demand for services catering to older adults. As the population ages, there will be a higher need for professionals trained to care for senior citizens, ranging from healthcare providers to legal advisors specializing in issues such as estate planning and elder rights. Workforce preparedness in these areas can reduce the strain on healthcare systems and ensure that older adults receive high-quality, specialized care [38-40]. Improving financial literacy among the younger generation concerning retirement planning and savings can also lessen future economic burdens. Young people educated on the importance of financial preparation for old age will likely enter their senior years with better economic security, reducing dependency on social welfare systems and easing fiscal pressures on governments [10,69,72].

Long-term societal gains from improved intergenerational harmony: Enhanced intergenerational harmony benefits society in several long-term ways. When younger and older generations interact positively, mutual respect and understanding are fostered, leading to more robust and cohesive communities. Intergenerational programs encouraging collaboration and communication can break down stereotypes and mistrust, replacing them with solidarity and shared goals [54]. Economic benefits also emerge from intergenerational harmony. Older adults can serve as mentors and advisors, transferring knowledge and skills to younger workers. This mentorship can improve job performance and innovation, boosting productivity and economic growth.

**Figure 5:**
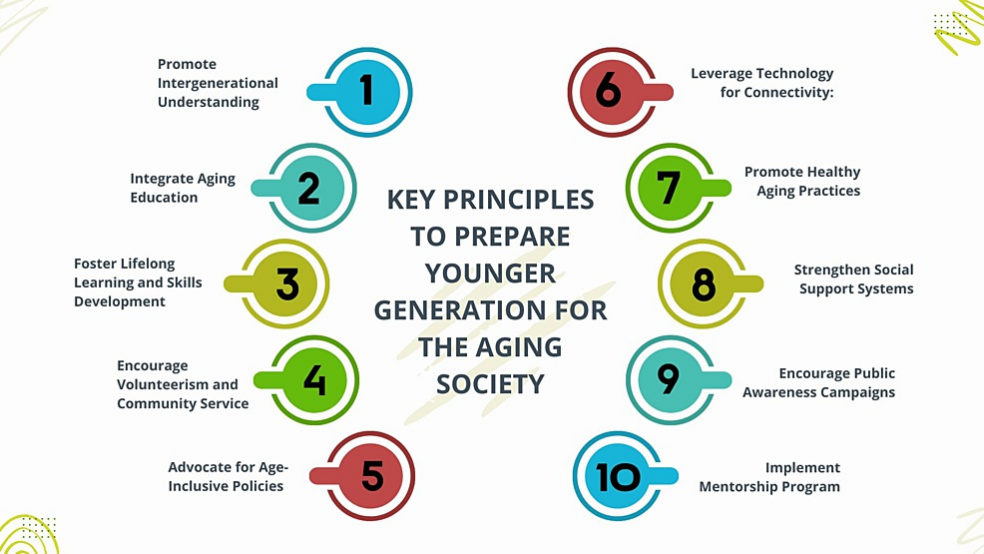
Key principles to prepare the younger generation for the aging society Image credit: Nor Faiza Mohd. Tohit

Furthermore, keeping older adults in the workforce for extended periods can contribute to financial stability, as their continued participation adds to the labor market and reduces the burden on pension systems [10,12,59]. Socially, intergenerational harmony can enhance family dynamics and community support systems. Families with multiple generations living together or near each other benefit from shared resources, stronger familial ties, and mutual caregiving [17-19]. Communities that value and promote intergenerational interaction tend to report higher levels of social trust and lower social isolation among older and younger members [56,73,74]. The fundamental principles in preparing the younger generation for the aging society are depicted in Figure 5.

Future research directions and areas for further exploration

While significant progress has been made in understanding the benefits of preparing younger generations for an aging society, further research is essential in several key areas. One critical direction for future research involves investigating how different cultural contexts influence the approach and effectiveness of intergenerational programs. Comparative studies could reveal best practices tailored to specific cultural settings, ensuring that interventions are culturally sensitive and effective globally [62]. Research should also explore the long-term impacts of integrating aging education into school curricula. Longitudinal studies tracking students who have participated in such programs could provide valuable insights into their long-term attitudes toward aging and their career choices related to eldercare. This data could inform educational policies and curriculum development to prepare future generations better [50,51].

**Figure 6:**
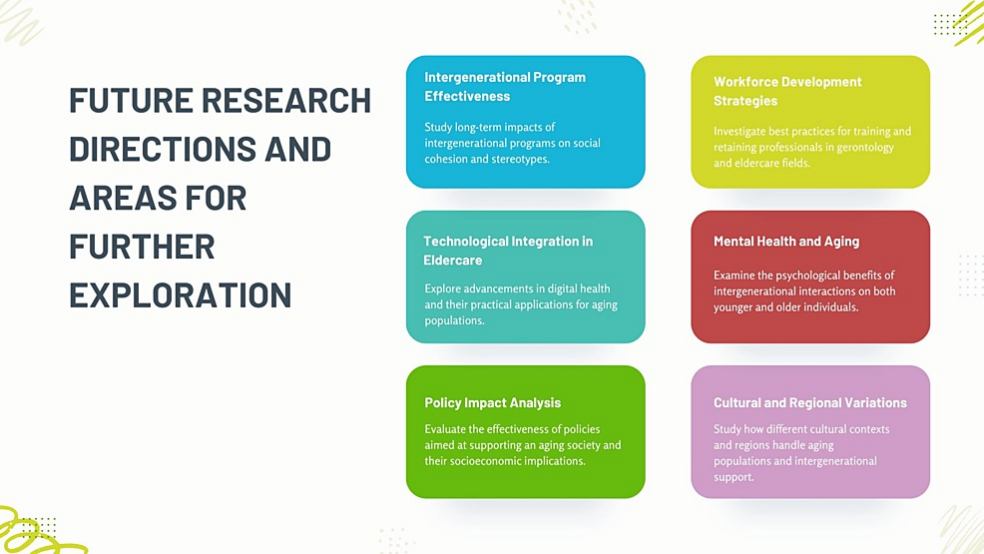
Future research directions and areas for further exploration Image credit: Nor Faiza Mohd. Tohit

Another area for exploration is the role of technology in enhancing intergenerational interactions. While initial studies have shown promise, deeper investigations are needed into how digital tools and platforms can facilitate sustained and meaningful generational exchanges. Understanding the barriers to technology adoption among older adults and the digital literacy of younger people will help design more effective technological solutions [75]. Lastly, studying the economic and policy impacts of a well-prepared younger generation on social systems, particularly healthcare and social security, is crucial. Simulation models predicting the financial outcomes of various intervention strategies could guide policymakers in making informed decisions to create sustainable and age-friendly societies [69]. The future research directions and areas for further exploration are shown in Figure 6.

## Conclusions

To prepare for an aging society, the younger generation must integrate aging education into school curricula and employ technology to bridge generational gaps. Addressing socio-economic and cultural barriers, combating ageism, and overcoming resistance within educational systems are vital steps. Such initiatives promote empathy, reduce stereotypes, and encourage intergenerational solidarity. Educators, policymakers, and communities must collaborate to incorporate aging-related content and intergenerational programs into educational frameworks. They should also promote policies that support lifelong learning and the active participation of older adults in society. By fostering mutual respect and understanding between generations, we can build a cohesive culture that values contributions from all age groups. This inclusive approach ensures a supportive environment where younger and older generations can thrive together, enhancing social cohesion and collective well-being.

## References

[1] United Nations. (2019 (2024). United Nations. World Population Prospects 2019: Highlights. Department of Economic and Social Affairs, Population Division. https://population.un.org/wpp/Publications/Files/WPP2019_10KeyFindings.pdf.

[2] Tamsma N, Costongs C (2018). Promoting health and well-being in the context of the United Nations Sustainable Development Agenda. Scand J Public Health.

[3] Shevelkova V, Mattocks C, Lafortune L (2023). Efforts to address the Sustainable Development Goals in older populations: a scoping review. BMC Public Health.

[4] Cristea M, Noja GG, Stefea P, Sala AL (2020). The impact of population aging and public health support on EU labor markets. Int J Environ Res Public Health.

[5] Rudnicka E, Napierała P, Podfigurna A, Męczekalski B, Smolarczyk R, Grymowicz M (2020). The World Health Organization (WHO) approach to healthy ageing. Maturitas.

[6] (2023). OECD/WHO (2020), "Ageing," in Health at a Glance: Asia/Pacific 2020: Measuring Progress Towards Universal Health Coverage, OECD Publishing, Paris. https://doi.org/10.1787/1ad1c42a-en.

[7] Jayawardhana T, Jayathilaka R, Nimnadi T, Anuththara S, Karadanaarachchi R, Galappaththi K, Dharmasena T (2023). The cost of aging: economic growth perspectives for Europe. PLoS One.

[8] Sahoo PM, Rout HS, Jakovljevic M (2023). Consequences of India's population aging to its healthcare financing and provision. J Med Econ.

[9] Moody E, Ganann R, Martin-Misener R (2022). Out-of-pocket expenses related to aging in place for frail older people: a scoping review. JBI Evid Synth.

[10] Nagarajan NR, Sixsmith A (2023). Policy initiatives to address the challenges of an older population in the workforce. Ageing Int.

[11] Barakovic Husic J, Melero FJ, Barakovic S (2020). Aging at work: a review of recent trends and future directions. Int J Environ Res Public Health.

[12] Quinn MM, Markkanen PK, Galligan CJ, Sama SR, Lindberg JE, Edwards MF (2021). Healthy aging requires a healthy home care workforce: the occupational safety and health of home care aides. Curr Environ Health Rep.

[13] Han Y, He Y, Lyu J, Yu C, Bian M, Lee L (2020). Aging in China: perspectives on public health. Glob Health J.

[14] Clancy RL, Fisher GG, Daigle KL, Henle CA, McCarthy J, Fruhauf CA (2020). Eldercare and work among informal caregivers: a multidisciplinary review and recommendations for future research. J Bus Psychol.

[15] Chen Z, Zhang X (2022). We were all once young: reducing hostile ageism from younger adults’ perspective. Front Psychol.

[16] Gans HM, Horhota M, Chasteen AL (2023). Ageism against older adults: how do intersecting identities influence perceptions of ageist behaviors?. J Appl Gerontol.

[17] Levy BR (2017). Age-stereotype paradox: opportunity for social change. Gerontologist.

[18] Burnette D, Ye X, Cheng Z, Ruan H (2021). Living alone, social cohesion, and quality of life among older adults in rural and urban China: a conditional process analysis. Int Psychogeriatr.

[19] Yu R, Leung G, Chan J, Yip BH, Wong S, Kwok T, Woo J (2021). Neighborhood social cohesion associates with loneliness differently among older people according to subjective social status. J Nutr Health Aging.

[20] Verhage M, Schuurman B, Lindenberg J (2021). How young adults view older people: exploring the pathways of constructing a group image after participation in an intergenerational programme. J Aging Stud.

[21] Kwong ANL, Yan ECW (2021). The role of quality of face-to-face intergenerational contact in reducing ageism: the perspectives of young people. J Intergener Relationsh.

[22] Ermer AE, York K, Mauro K (2021). Addressing ageism using intergenerational performing arts interventions. Gerontol Geriatr Educ.

[23] Andrew S (2021). Toward interventions to reduce internalized ageism. J Human Behav Soc Environ.

[24] Lytle A, Nowacek N, Levy SR (2020). Instapals: reducing ageism by facilitating intergenerational contact and providing aging education. Gerontol Geriatr Educ.

[25] Mitzner TL, Savla J, Boot WR, Sharit J, Charness N, Czaja SJ, Rogers WA (2019). Technology adoption by older adults: findings from the PRISM trial. Gerontologist.

[26] Czaja SJ, Boot WR, Charness N, Rogers WA, Sharit J (2018). Improving social support for older adults through technology: findings from the prism randomized controlled trial. Gerontologist.

[27] Thangavel G, Memedi M, Hedström K (2022). Customized information and communication technology for reducing social isolation and loneliness among older adults: scoping review. JMIR Ment Health.

[28] Zhao M, Yang F, Zhang Y (2022). The power of culture: the gendered impact of family structures and living arrangements on social networks of Chinese older adults. Ageing Soc.

[29] Seeman TE, Crimmins E (2001). Social environment effects on health and aging: integrating epidemiologic and demographic approaches and perspectives. Ann N Y Acad Sci.

[30] Arksey H, O’Malley L (2005). Scoping studies: towards a methodological framework. Int J Soc Res Method.

[31] Levac D, Colquhoun H, O'Brien KK (2010). Scoping studies: advancing the methodology. Implement Sci.

[32] Aguilera-Hermida AP, Anderson EA, Negrón VA (2020). Intergenerational activities that promote engaging conversations are preferred among young and older adults. J Intergener Relationsh.

[33] Barbra T (2016). Intergenerational programs to promote active aging: the experiences and perspectives of older adults. Activ Adapt Aging.

[34] Lytle A, Levy SR (2019). Reducing ageism: education about aging and extended contact with older adults. Gerontologist.

[35] Boulton-Lewis GM (2010). Education and learning for the elderly: why, how, what. Educ Gerontol.

[36] Masud T, Ogliari G, Lunt E (2022). A scoping review of the changing landscape of geriatric medicine in undergraduate medical education: curricula, topics and teaching methods. Eur Geriatr Med.

[37] Naidoo K, Van Wyk J (2020). Preparing medical graduates to care for geriatric patients: a case study of the undergraduate medical curriculum at a South African university. S Afr Fam Pract (2004).

[38] Sabzwari SR, Bhanji S, Zuberi RW (2011). Integration of geriatrics into a spiral undergraduate medical curriculum in Pakistan: evaluation and feedback of third-year medical students. Educ Health (Abingdon).

[39] Naughton C, O'Shea KL, Hayes N (2019). Incentivising a career in older adult nursing: the views of student nurses. Int J Older People Nurs.

[40] Diachun LL, Hillier LM, Stolee P (2006). Interest in geriatric medicine in Canada: how can we secure a next generation of geriatricians?. J Am Geriatr Soc.

[41] Kruse C, Fohn J, Wilson N, Nunez Patlan E, Zipp S, Mileski M (2020). Utilization barriers and medical outcomes commensurate with the use of telehealth among older adults: systematic review. JMIR Med Inform.

[42] Turjamaa R, Pehkonen A, Kangasniemi M (2019). How smart homes are used to support older people: an integrative review. Int J Older People Nurs.

[43] Eun-Kyoung LO, Kim DH (2019). Bridging the digital divide for older adults via intergenerational mentor-up. Res Social Work Prac.

[44] Rochmawati E, Kamilah F, Iskandar AC (2022). Acceptance of e-health technology among older people: a qualitative study. Nurs Health Sci.

[45] Anina V, Schirmer W, Geerts N, Mortelmans D (2023). How “basic” is basic digital literacy for older adults? Insights from digital skills instructors. Front Educ.

[46] Francis J, Kadylak T, Makki TW, Rikard RV, Cotten SR (2018). Catalyst to connection: when technical difficulties lead to social support for older adults. Am Behav Sci.

[47] Zhong S, Lee C, Foster MJ, Bian J (2020). Intergenerational communities: a systematic literature review of intergenerational interactions and older adults' health-related outcomes. Soc Sci Med.

[48] Wu VX (2021). Health promotion in the community via an intergenerational platform: Intergenerational e-Health Literacy Program (I-HeLP). Health Promotion in Health Care - Vital Theories and Research.

[49] Vacca R, Bianchi F (2024). Diversity, integration, and variability of intergenerational relationships in old age: new insights from personal network research. Soc Sci Res.

[50] Wong BL, Siepmann I, Rangan A (2021). Involving young people in healthy ageing: a crucial facet to achieving the decade of healthy ageing (2021-2030). Front Public Health.

[51] Clemson L (2022). Relevance, resilience, and ageism: a bright future for occupational therapy and healthy ageing, Sylvia Docker Lecture 2021. Aust Occup Ther J.

[52] Sanchez M (2024). Sánchez M, Butts DM, Hatto-Yeo A, et al. Intergenerational programs Towards a society for all ages. Social Studies Collection. No.23. https://generationsworkingtogether.org/downloads/53aabc130d1c6-IG%20programmes%20-%20towards%20a%20society%20for%20all%20ages%20vol23_en.pdf.

[53] Le AH, Billett S (2022). Lifelong learning and adult education in Japan. Aust J Adult Learn.

[54] (2024). Generations United. https://www.gu.org/.

[55] Ng R, Indran N (2023). Granfluencers on TikTok: factors linked to positive self-portrayals of older adults on social media. PLoS One.

[56] Fowler Davis S, Benkowitz C, Holland C, Gow A, Clarke C (2024). A scoping review on the opportunities for social engagement and cognitive frailty in older adults. Public Health Rev.

[57] Dunkle RE, Mikelthun BG (1983). Intergenerational programming: an Adopt-a-Grandparent program in a retirement community. Activ Adapt Aging.

[58] Martinson M, Minkler M (2006). Civic engagement and older adults: a critical perspective. Gerontologist.

[59] Rémillard-Boilard S, Buffel T, Phillipson C (2020). Developing age-friendly cities and communities: eleven case studies from around the world. Int J Environ Res Public Health.

[60] Amuthavalli Thiyagarajan J, Mikton C, Harwood RH (2022). The UN Decade of healthy ageing: strengthening measurement for monitoring health and wellbeing of older people. Age Ageing.

[61] Carroll J, Hopper L, Farrelly AM, Lombard-Vance R, Bamidis PD, Konstantinidis EI (2021). A scoping review of augmented/virtual reality health and wellbeing interventions for older adults: redefining immersive virtual reality. Front Virtual Real.

[62] Charise A, Pang C, Khalfan KA (2022). What is intergenerational storytelling? Defining the critical issues for aging research in the humanities. J Med Humanit.

[63] Talal M, Zaidan AA, Zaidan BB (2019). Smart home-based IoT for real-time and secure remote health monitoring of triage and priority system using body sensors: multi-driven systematic review. J Med Syst.

[64] Phang JK, Kwan YH, Yoon S, Goh H, Yee WQ, Tan CS, Low LL (2023). Digital intergenerational program to reduce loneliness and social isolation among older adults: realist review. JMIR Aging.

[65] Welch V, Ghogomu ET, Barbeau VI (2023). Digital interventions to reduce social isolation and loneliness in older adults: an evidence and gap map. Campbell Syst Rev.

[66] Ventura S, Badenes-Ribera L, Herrero R, Cebolla A, Galiana L, Baños R (2020). Virtual reality as a medium to elicit empathy: a meta-analysis. Cyberpsychol Behav Soc Netw.

[67] Longhini J, Rossettini G, Palese A (2021). Massive open online courses for nurses' and healthcare professionals' continuous education: a scoping review. Int Nurs Rev.

[68] Ng R, Indran N (2024). Innovations for an aging society through the lens of patent data. Gerontologist.

[69] Wildman JM, Goulding A, Moffatt S, Scharf T, Stenning A (2022). Intergenerational equity, equality and reciprocity in economically and politically turbulent times: narratives from across generations. Ageing Soc.

[70] Duflos M, Giraudeau C (2022). Using the intergenerational solidarity framework to understand the grandparent-grandchild relationship: a scoping review. Eur J Ageing.

[71] Liou AL, Literat I (2020). “We need you to listen to us”: youth activist perspectives on intergenerational dynamics and adult solidarity in youth movements. Int J Commun.

[72] Lusardi A (2019). Financial literacy and the need for financial education: evidence and implications. Swiss J Econ Stat.

[73] Krzeczkowska A, Spalding DM, McGeown WJ, Gow AJ, Carlson MC, Nicholls LA (2021). A systematic review of the impacts of intergenerational engagement on older adults' cognitive, social, and health outcomes. Ageing Res Rev.

[74] Md Fadzil NH, Shahar S, Rajikan R (2022). A scoping review for usage of telerehabilitation among older adults with mild cognitive impairment or cognitive frailty. Int J Environ Res Public Health.

[75] Keating N (2022). A research framework for the United Nations Decade of Healthy Ageing (2021-2030). Eur J Ageing.

